# Connecting Cohorts to Diminish Alzheimer’s Disease (CONCORD-AD): A Report of an International Research Collaboration Network

**DOI:** 10.3233/JAD-210525

**Published:** 2022-01-04

**Authors:** Valory N. Pavlik, Samantha C. Burnham, Joseph S. Kass, Catherine Helmer, Sebastian Palmqvist, Maria Vassilaki, Jean-François Dartigues, Oskar Hansson, Colin L. Masters, Karine Pérès, Ronald C. Petersen, Erik Stomrud, Lesley Butler, Preciosa M. Coloma, Xavier M. Teitsma, Rachelle Doody, Mary Sano, for the CONCORD-AD investigators

**Affiliations:** a Department of Neurology, Baylor College of Medicine, Houston, TX, USA; b The Australian eHealth Research Centre, CSIRO Health and Biosecurity, Melbourne, VIC, Australia; c University of Bordeaux, Inserm, Bordeaux Population Health Research Center, UMR, Bordeaux, France; d Clinical Memory Research Unit, Lund University, Lund, Sweden; e Department of Quantitative Health Sciences, Division of Epidemiology, Mayo Clinic, Rochester, MN, USA; f Department of Neurology, Memory Consultation, Bordeaux University Hospital, Bordeaux, France; g Memory Clinic, Skåne University Hospital, Malmö, Sweden; h The Florey Institute and The University of Melbourne, Parkville, VIC, Australia; i Department of Neurology, Mayo Clinic, Rochester, MN, USA; j Product Development Personalised Health Care – Data Science, F. Hoffmann-La Roche Ltd, Basel, Switzerland; k Product Development Neuroscience, F. Hoffmann-La Roche Ltd, Basel, Switzerland; l Product Development Neuroscience, Genentech, Inc., South San Francisco, CA, USA; m Department of Psychiatry, Alzheimer’s Disease Research Center, Icahn School of Medicine at Mount Sinai, New York, NY, USA; n James J. Peters VA Medical Center, Bronx, NY, USA

**Keywords:** Alzheimer’s disease, biomarkers, cognitive function, cohort, CONCORD-AD network, dementia, observational study, population characteristics

## Abstract

Longitudinal observational cohort studies are being conducted worldwide to understand cognition, biomarkers, and the health of the aging population better. Cross-cohort comparisons and networks of registries in Alzheimer’s disease (AD) foster scientific exchange, generate insights, and contribute to the evolving clinical science in AD. A scientific working group was convened with invited investigators from established cohort studies in AD, in order to form a research collaboration network as a resource to address important research questions. The Connecting Cohorts to Diminish Alzheimer’s Disease (CONCORD-AD) collaboration network was created to bring together global resources and expertise, to generate insights and improve understanding of the natural history of AD, to inform design of clinical trials in all disease stages, and to plan for optimal patient access to disease-modifying therapies once they become available. The network brings together expertise and data insights from 7 cohorts across Australia, Europe, and North America. Notably, the network includes populations recruited through memory clinics as well as population-based cohorts, representing observations from individuals across the AD spectrum. This report aims to introduce the CONCORD-AD network, providing an overview of the cohorts involved, reporting the common assessments used, and describing the key characteristics of the cohort populations. Cohort study designs and baseline population characteristics are compared, and available cognitive, functional, and neuropsychiatric symptom data, as well as the frequency of biomarker assessments, are summarized. Finally, the challenges and opportunities of cross-cohort studies in AD are discussed.

## BACKGROUND

Alzheimer’s disease (AD) is the leading cause of dementia [1]. It is a progressive and, as of yet, incurable neurodegenerative disease that has devastating consequences for the lives of affected individuals and their families. Fifty million people worldwide were living with dementia in 2018, and this number is expected to more than triple to reach 152 million by 2050 [2]. Dementia is a leading cause of morbidity, mortality, and disability, especially among the elderly [3]. Economic estimates have suggested the total annual cost of dementia worldwide was approximately 1 trillion US dollars in 2018, and this cost is expected to double by 2030 [2]. Without intervention, AD will devastate public health resources and overwhelm the current healthcare infrastructure on a global scale. Postponing dementia onset by even just one year could result in nine million fewer cases worldwide than predicted for 2050 [4].

To mitigate the impact of AD on both the individual and society, novel disease-modifying therapies are urgently needed to treat the disease or delay its onset. The development of new therapies must also be accompanied by careful analysis of the populations most affected by AD and the potential impact of new treatment options on clinical practice. To this end, a variety of longitudinal observational studies are being conducted around the world to assess cognition, biomarkers, and the health of the aging population. These range from population-based community studies monitoring participants with and without dementia at enrollment, to studies of older adults from memory clinics with complaints of cognitive decline. International collaborations that leverage diverse clinical data and scientific expertise could improve our understanding of disease etiology and the natural history of AD, generate insights to inform the design of preventive and therapeutic trials, and inform health-service planning for optimal early detection of cognitive decline and patient access to new disease-modifying therapies as they become available.

Cross-cohort analyses and networks of registries in AD have previously been established, each with its own objectives, e.g., to facilitate clinical trial recruitment [5], monitor changes in the incidence of dementia [6, 7], identify risk factors, improve early diagnosis, or address specific research questions [8–10]. In addition, publicly available collaborative resources such as the Global Alzheimer’s Association Interactive Network (GAAIN) research platform [11] have been set up to allow researchers to access data being collected across the globe and to promote consistency and comparability across cohort analyses. The newly assembled Connecting Cohorts to Diminish Alzheimer’s Disease (CONCORD-AD) network was created to foster scientific exchange, generate insights, and contribute to the evolving clinical science in AD.

## DEVELOPMENT OF THE INTERNATIONAL CONCORD-AD COLLABORATION NETWORK

In August 2016, a group of investigators from around the world, representing observational prospective cohorts of individuals across the AD continuum, convened to form a scientific working group; its aim, to establish a research collaboration network as a resource to address important research questions related to the natural history and burden of AD. A group of investigators from established longitudinal cohort studies in AD were invited to participate, representing a diverse range of studies from around the globe. The resultant CONCORD-AD network brought together expertise and data insights from seven cohorts across Australia, Europe, and North America. Notably, the network includes populations recruited through memory clinics and population-based cohorts, bringing together observations from individuals across the AD spectrum—from cognitively unimpaired (CU) people with or without subjective cognitive decline (SCD), to those with mild cognitive impairment (MCI), through to those with AD, as well as other types of dementia. Although each cohort was established with different objectives, this network provides the opportunity to address novel research questions across the cohorts and to leverage disease area expertise among the investigators.

In this article, we introduce the CONCORD-AD network, provide an overview of the cohorts involved, report the common assessments used in the cohorts, and describe the key characteristics of the cohort populations. We show examples of knowledge gaps already being addressed by each cohort and highlight the diversity, similarities, and complementarity among these different data sources. We also provide recommendations on how such collaborations may evolve in the future, and the challenges and opportunities they can present.

## OVERVIEW OF THE CONCORD-AD PARTICIPATING COHORTS

The seven cohorts participating in the CONCORD-AD network represent data from more than 20,000 individuals across the disease spectrum, with a maximum follow-up period of 27 years. The key characteristics of the different cohorts are summarized in Table 1, and the definitions used for CU, MCI, or AD cognitive subgroups are listed in Supplementary Table 1 [44–53]. Brief summaries of the cohort study design and their previous applications in research follow. The required ethical approval processes were completed as appropriate within the individual studies.

**Table 1:** Study and baseline characteristics of the cohorts included in the CONCORD-AD network at study enrollment

	AIBL	Baylor	BioFINDER-1	MCSA	PAQUID	3C Bordeaux	AMI
Study participants^a^, *n* (%)
CU	1,190 (66)	76 (3)	834 (68)	4,055 (86)	3,575^b^ (98)	2,027^b^ (97)	877^b^ (91)
MCI	295 (16)	413 (13)	292 (24)	562 (12)	N/A	N/A	N/A
AD	322 (18)	2,622 (84)	93 (8)	79^d^ (2)	79 (2)	59 (3)	86^e^ (9)
Country	Australia	USA	Sweden	USA	France	France	France
Setting	Memory clinic and population-based	Memory clinic	Memory clinic and population-based	Population-based	Population-based	Population-based	Population-based
Mean follow-up (y)^c^	∼4.5	∼3	∼5	∼5	12.0	12.7	5.5
Attrition rate per year (%)	8.7	5.1	4.8	7.5	3.6	4.3	5.9
Ongoing follow-up (yes/no)	Yes	Yes	Yes	Yes	No	Yes	Yes
Age > 70 y, *n* (%)	1,001 (55)	2,158 (68)	921 (59)	3,326 (71)	2,677 (71)	1,636 (78)	786 (78)
Male, *n* (%)	768 (43)	1,240 (39)	740 (48)	2,388 (51)	1577 (42)	816 (39)	626 (62)
> 12 y of education, *n* (%)	895 (49)	1,883 (59)	584 (38)	3,009 (64)	387 (10)	779^f^ (37)	50^g^ (5)
*APOE* *ɛ*4 carrier, *n* (%)	547 (30)	1,427 (46)	587 (38)	1,299 (28)	151^h^ (23)	365^i^ (20)	114^j^ (18)

AD, Alzheimer’s disease; AIBL, Australian Imaging, Biomarkers & Lifestyle Flagship Study of Ageing; AMI, AGRICA-MSA-Institut fédératif de recherche en santé publique/Aging Multidisciplinary Investigation; *APOE* *ɛ*4, apolipoprotein E *ɛ*4 allele; Baylor, Alzheimer’s Disease and Memory Disorders Center at Baylor College of Medicine; BioFINDER-1, Biomarkers For Identifying Neurodegenerative Disorders Early and Reliably; CU, cognitively unimpaired; MCI, mild cognitive impairment; MCSA, Mayo Clinic Study of Aging; PAQUID, Personnes Agées QUID; 3C Bordeaux, Three-City Study. ^a^Selected cohorts also enrolled participants with other types of dementia (not displayed in the present report); ^b^Non-demented; ^c^Mean follow-up period at the time individual cohort data were submitted for inclusion within the CONCORD-AD analyses; ^d^AD or other dementia; ^e^AD or AD plus another form of dementia; ^f^Data available for 2,099 participants; ^g^Data available for 999 participants; ^h^Data available for 646 participants; ^i^Data available for 1,866 participants; ^j^Data available for 647 participants.

### The Australian Imaging, Biomarker & Lifestyle Flagship Study of Ageing (AIBL) Cohort

The AIBL study was established in 2006 in Australia and had recruited 1,807 participants aged≥60 years at the time the CONCORD-AD network was established (CU, *n* = 1,190; MCI, *n* = 295; AD, *n* = 322; June 1, 2018 analysis) through treating physicians at memory clinics and community appeals [12]. Assessments took place at central locations in Melbourne and Perth, depending on whether participants underwent brain imaging, and where they lived; a small number were also assessed at home by AIBL staff. The AIBL cohort has given rise to diverse research publications and further studies, including imaging studies exploring the relationship between aggregated amyloid-β (Aβ) and gray matter atrophy [13], studies exploring the application of new research frameworks and analytical techniques in the AIBL cohort [14–16], cognition-function studies [17], and the potential for introducing physical activity interventions in participants at risk for AD or with subjective memory complaints [18].

### The Alzheimer’s Disease and Memory Disorders Center at Baylor College of Medicine (Baylor) cohort

Since 1989, the US-based Baylor study has recruited 3,181 participants aged≥60 years (CU, *n* =76; MCI, *n* = 413; AD, *n* = 2,622; November–December 2017 analysis) referred to the Baylor memory clinic [19]. Self-referred and physician-referred participants with memory complaints were recruited at the Houston, TX, USA clinic and underwent various laboratory tests, including neuroimaging and psychometric assessments. Previous publications based on these data have evaluated the relationship between initial progression rate and subsequent longitudinal progression of cognitive and functional measures [20], prevalence, predictors, and clinical outcomes of different cognitive profiles within AD [21–25], and overall survival in participants with probable AD [26].

### The Swedish Biomarkers for Identifying Neurodegenerative Disorders Early and Reliably (BioFINDER-1) Study

The Swedish BioFINDER-1 study started to recruit participants in 2008 to address the knowledge gaps in early detection of underlying pathologies and subsequent disease mechanisms in AD and Parkinson’s disease [27], and has recruited 1,554 participants, including 1,219 in the cohort for AD-focused research (CU, *n* = 834; MCI, *n* = 292; AD, *n* = 93; as of March 16, 2020). Participants aged≥60 years with mild cognitive symptoms, dementia, and Parkinsonian symptoms were recruited through memory and neurology clinics at several participating centers in Sweden; healthy CU elderly participants were recruited through a pre-existing population-based community cohort [28]. BioFINDER-1 data have been used to explore the use and accuracy of biomarkers for diagnosing AD [29, 30] and how this could be further optimized [31–33], and also to evaluate the relationship between biomarkers and pathology [34, 35].

### The Mayo Clinic Study of Aging (MCSA)

The Mayo Clinic Study of Aging (MCSA) is a prospective population-based cohort study (most participants ≥50 years old) established in 2004 in Olmsted County (MN), USA, to investigate the prevalence and incidence of MCI, cognitive aging, as well as the risk factors and conversion rates for MCI and dementia (CU, *n* = 4,055; MCI, *n* = 562; dementia, *n* = 79; as of February 28, 2018 analysis). MCSA examines the prevalence of cognitive impairment, dementia and vascular biomarkers [36] and their association with cognitive outcomes [37–41] as well as the development of risk scores to predict biomarker and cognitive outcomes [42].

### Personnes Agées QUID (PAQUID)

The PAQUID study recruited older adults, aged ≥65 years and living at home, from the general population in Southwestern France in order to study normal and pathological cerebral aging [43] (non-demented, *n* = 3,675; AD, *n* = 79; non-AD dementia, *n* = 23 per baseline assessment; based on prevalent cases at inclusion in 1988–1990; last visits: October 2015–July 2016). This study has the longest follow-up time of the included cohorts, established in 1988 with as much as 30 years of follow-up. PAQUID data have been used in epidemiological studies to estimate dementia and cognitive impairment prevalence and incidence trends together with the 3C Bordeaux and AMI cohorts [44, 45], to evaluate relationships between social/environmental factors and cognitive decline and dementia [46], to evaluate factors affecting healthcare resource use in participants with dementia (with 3C study and AMI cohorts) [47], and to evaluate functional and cognitive trajectories of decline before dementia and brain vulnerability [48–50].

### Three-City Study Bordeaux (3C Bordeaux)

The 3C Study is a population-based, longitudinal study that started in 1999 and has a maximum follow-up time of 17 years. The study recruited participants aged ≥65 years from the general populations of three cities in France: Bordeaux, Dijon, and Montpellier [51], including 2,104 participants from the Bordeaux center, data from whom are part of the CONCORD-AD network (non-demented, *n* = 2,027; AD, *n* = 59; non-AD dementia, *n* = 18; based on prevalent cases at inclusion 1999–2001). Epidemiological studies using this cohort have evaluated the associations between depressive symptoms, co-morbidities such as diabetes, and dietary factors on the risk of dementia and AD [52–57].

The COGICARE study is an ancillary substudy of the Three-City (3C) study in Bordeaux and Montpellier centers. COGICARE was designed to characterize the natural history of cognitive and functional decline around dementia through close follow-up of subjects at three different stages: AD, MCI, or cognitively normal. It began at the 10-year follow-up of the 3C study and included 467 participants who underwent cognitive and functional assessments every 6 months, for up to 24 months, in addition to their 3C follow-ups. The protocol of the COGICARE study was approved by the Ethics Committee of Sud-Méditerranée III (France) and written informed consent was obtained for each participant.

### Aging Multidisciplinary Investigation (AMI)

The AMI study was established in 2007 to study health and aging in elderly farmers in rural areas of France [58] (maximum follow-up: 10 years). The study recruited 1,002 participants aged≥65 years from the Farmer Health Insurance rolls who had retired from agriculture (non-demented, *n* = 877; AD, *n* = 86; non-AD dementia, *n* = 39; based on prevalent cases at inclusion 2007–2009; last visits: April 2014–May 2015). In addition to collaborative analyses with the PAQUID and 3C Bordeaux cohorts, has allowed researchers to further investigate dementia screening in the elderly [59] and putative associations between the C–C Motif Chemokine Ligand 11 biomarker and cognitive status in older participants [60].

## THE COMPLEMENTARITY AND DIVERSITY OF THE CONCORD-AD NETWORK

### Patient demographics and characteristics

As a consequence of the varied study designs, recruitment protocols, and inclusion/exclusion criteria of the different cohorts, there were differences in the demographic make-up of the participants enrolled in each of these studies (Table 1). Cohorts in CONCORD-AD include participants recruited either from memory clinics (Baylor study), the community population (MCSA, PAQUID, 3C Bordeaux, and AMI), or both memory clinics and the general population (AIBL and BioFINDER-1). The percentage of participants aged > 70 years ranged from 55% (AIBL) to 78% (AMI and 3C Bordeaux) and the percentage of male participants varied from 39% in Baylor and 3C Bordeaux to 62% in AMI, where the high percentage of males in an aging population can be attributed to the inclusion criteria requiring participants to be retired from agriculture and affiliated to the Health Insurance under their own name (in some regions women were commonly affiliated under their husband’s name). Educational background varied markedly, with only 5% of the AMI rural cohort completing > 12 years of education, compared with 64% in MCSA. The prevalence of apolipoprotein E *ɛ*4 allele (*APOE* *ɛ*4) carriers varied widely as well, from 18% (AMI) to 46% (Baylor) in keeping with the higher percentage of people with AD at the Baylor site.

### Assessments available for cross-cohort comparison in CONCORD-AD

**Fig. 1:**
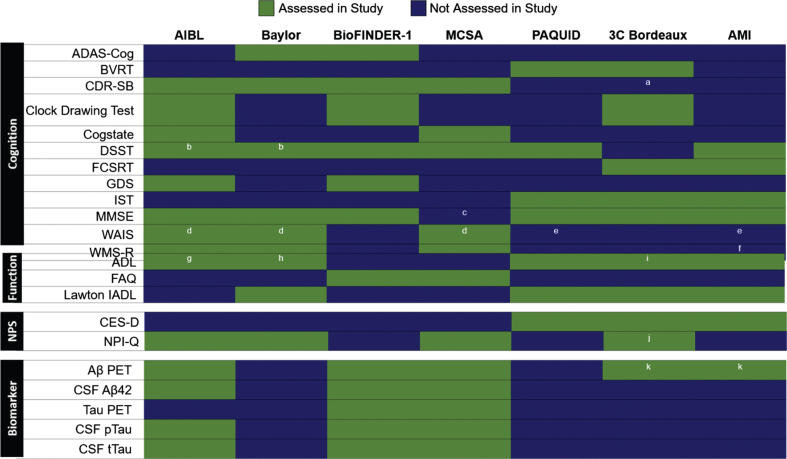
Assessments conducted in at least two of the CONCORD-AD cohorts. Includes assessments performed in cohort studies in different geographic regions. Aβ, amyloid-β; AD, Alzheimer’s disease; ADAS-Cog, Alzheimer’s Disease Assessment Scale–Cognitive subscale; ADL, Activities of daily living; AIBL, Australian Imaging, Biomarkers & Lifestyle Flagship Study of Ageing; AMI, AGRICA-MSA-Institut fédératif de recherche en santé publique/Aging Multidisciplinary Investigation; BioFINDER-1, Biomarkers For Identifying Neurodegenerative Disorders Early and Reliably; BVRT, Benton Visual Retention test; CDR-SB, Clinical Dementia Rating–Sum of Boxes; CES-D, Centre for Epidemiologic Studies Depression; CSF, cerebrospinal fluid; CU, cognitively unimpaired; DSST, Digit Symbol Substitution Test; FAQ, Functional Assessment Questionnaire; FCSRT, Free and Cued Selective Reminding Test; GDS, Geriatric Depression Scale; IADL, Instrumental Activities of Daily Living; IST, Isaacs Set Test; MCI, mild cognitive impairment; MCSA, Mayo Clinic Study of Aging; MINI, Mini-International Neuropsychiatric Interview; MMSE, Mini-Mental State Examination; ND, non-demented; NPI-Q, Neuropsychiatric Inventory Questionnaire; NPS, neuropsychiatric symptoms; PET, positron emission tomography; pTau, phosphorylated tau; tTau, total tau; WAIS-III, Wechsler Adult Intelligence Scale–Third Edition; WAIS-R, Wechsler Adult Intelligence Scale–Revised; WMS-R, Wechsler Memory Scale–Revised; 3C Bordeaux, Three-City Study. ^a^Used in the COGICARE sub-study of 3C Bordeaux in all participants with dementia; ^b^DSST can be derived from the WAIS-R used in Baylor and from WAIS-III in AIBL; ^c^MMSE score can be derived from the Short Test of Mental Status used in MCSA; ^d^WAIS-R in Baylor, WAIS-III in AIBL; ^e^Wechsler similarities test used; ^f^Wechsler story memory test; ^g^ADL Inventory; ^h^Lawton and Brody instrumental ADL Scales; ^i^Katz scale; ^j^Short form used in the COGICARE sub-study of 3C; ^k^Amyloid-PET available only on a subsample at the follow-up.

A wide variety of assessments were used to monitor cognition, function, neuropsychiatric symptoms (NPS), and biomarkers in the CONCORD-AD cohorts (Fig. 1). The most common cognitive assessment was the Mini-Mental State Examination (MMSE), which was used in all cohorts except the MCSA, which included the Short Test of Mental Status from which MMSE score can be derived [61, 62]. The Clinical Dementia Rating – Sum of Boxes (CDR-SB), a commonly used metric in clinical trials, was assessed in four of the cohorts, while the Digit Symbol Substitution Test (DSST), Isaacs Set Test (IST), and Wechsler Memory Scale–Revised (WMS-R) were each applied in three cohorts. An Activities of Daily Living (ADL) questionnaire was the most commonly used functional assessment, and three different functional assessments were used in ≥2 cohorts (Fig. 1). The Lawton Instrumental ADL (IADL) scale was assessed in the Baylor and French cohorts. Similarly, three distinct inventories were used to measure neuropsychiatric symptoms in ≥2 cohorts, of which the self-reported Center for Epidemiologic Studies – Depression (CES-D) scale was only used in the French cohorts (Fig. 1).

### Cognitive impairment across the AD spectrum

**Fig. 2:**
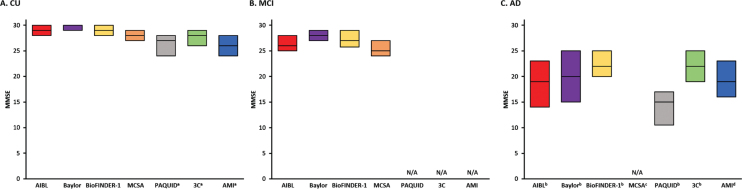
Comparison of baseline MMSE scores across the cohorts in (A) CU, (B) MCI, and (C) AD participants. Note that for population-based cohorts, AD at baseline are prevalent cases, and not incident cases or cases recently referred to a memory clinic. AD, Alzheimer’s disease; AIBL, Australian Imaging, Biomarkers & Lifestyle Flagship Study of Ageing; AMI, AGRICA-MSA-Institut fédératif de recherche en santé publique/Aging Multidisciplinary Investigation; Baylor, Alzheimer’s Disease and Memory Disorders Center at Baylor College of Medicine; BioFINDER-1, Biomarkers For Identifying Neurodegenerative Disorders Early and Reliably; CU, cognitively unimpaired; MCSA, Mayo Clinic Study of Aging; MMSE, Mini-Mental State Examination; ND, no data; PAQUID, Personnes Agées QUID; 3C Bordeaux, Three-City Study. ^a^The PAQUID, 3C Bordeaux and AMI studies scores are presented of non-demented participants; ^b^The AIBL, Baylor, and BioFINDER-1, data presented is specific to AD dementia (clinically defined AD or biomarker-confirmed AD; BioFINDER-1 includes AD with other pathologies where AD is the dominant etiology); ^c^MCSA included dementia of any cause; ^d^3C Bordeaux, PAQUID, and AMI included AD or AD plus another form of dementia (with another type of lesion or atypical clinical presentation).

MMSE scores at baseline were similar in CU participants from AIBL, Baylor, and BioFINDER-1 cohorts (median range 29–30). Notably, these studies all included participants from memory clinics. Participants in the community-based MCSA, 3C Bordeaux, PAQUID, and AMI (non-demented) cohorts had a wider range of scores (median range 26–28) (Fig. 2A). As expected, in both clinic- and community-based cohorts, there was progressive worsening in MMSE scores in the MCI group (median range 25–28) and AD dementia participants (median range 15–22) (Fig. 2B, C). In the four cohorts that also utilized CDR-SB to monitor disease progression, baseline CDR-SB scores for CU individuals were low, with a median score of 0 for all cohorts except the Baylor study (median 1, Supplementary Figure 1). As with the MMSE, there was progressive worsening in the MCI (median range 0.5–1.5) and AD dementia groups (median both 6) (Supplementary Figure 1B, 1C).

### Functional impairment measures varied across the cohorts

A variety of scales were used to assess functional impairment in the CONCORD-AD network; therefore, direct score comparisons of functional impairments were not possible in this report. Baseline functional assessments in the COGICARE ancillary study of the 3C cohort showed that among participants living in the community, those with dementia had higher rates of moderate (4-IADL score = 3; 14.4%) or severe IADL disability (4-IADL score = 4; 64.0%), compared with 5.2% and 2.6%, respectively in those with MCI (MCI status retrospectively assigned for this specific analysis). The ADL impairment was associated with increased risk of moderate-to-severe caregiver burden. Functional decline was evaluated in the MCSA (measured with the Functional Activities Questionnaire [FAQ] and the CDR-SB functional domains) and Baylor (IADL and the Lawton and Brody Physical Self-Maintenance Scale [PSMS]) studies. In the MCSA, more pronounced functional limitations and decline correlated with elevated brain amyloid and neurodegeneration [63, 64]. Interestingly in a temporal assessment of 5-year functional decline rate of individuals with probable AD from the Baylor study, more recent cohorts (2005–2009) had a lower rate of functional decline in PSMS (but not IADL) than earlier participants (1994–1999 and 2000–2004).

### Occurrence of neuropsychiatric symptoms across different stages of AD

**Fig. 3:**
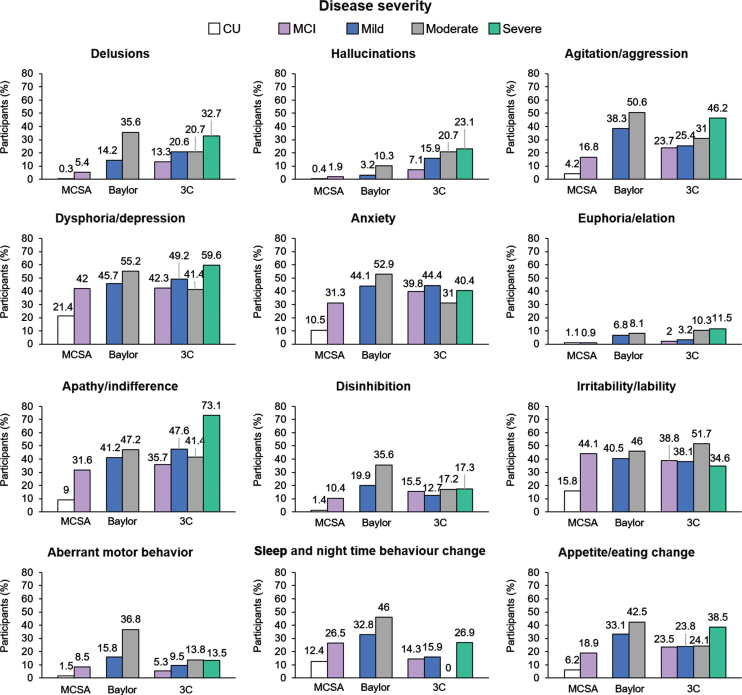
Frequency of NPS, as assessed by NPI-Q, and displayed by disease stage in the MCSA^a^, 3C^b^, and Baylor cohorts^c^. AD, Alzheimer’s Disease; MCSA, Mayo Clinic Study of Aging; Baylor, Alzheimer’s Disease and Memory Disorders Center at Baylor College of Medicine; CU, cognitively unimpaired; MCI, mild cognitive impairment; 3C, Three-City Study; NPI-Q, Neuropsychiatric Inventory–Questionnaire; NPS, Neuropsychiatric symptoms. ^a^In non-demented and≥50 years of age participants, with a concurrent, valid NPS assessment and amyloid positron emission tomography (PET) neuroimaging. MCSA data have been reported previously [65]; ^b^Results presented from COGICARE, a sub-study of 3C; ^c^Data were not available from the other cohorts.

Data on NPS collected using the Neuropsychiatric Inventory Questionnaire (NPI-Q) were available from MCSA (in those with positron emission tomography [PET] imaging for current report), COGICARE sub-study of 3C, and Baylor cohorts. Dysphoria/depression, anxiety, apathy/indifference, and irritability/lability were the most common NPS overall (Fig. 3) [65]. Psychotic symptoms such as hallucinations and euphoria/elation were less common overall. The frequency of NPS generally increased with severity of disease (Fig. 3). In the MCSA cohort, NPS were reported at a higher rate in the MCI group than CU individuals for all NPS, except euphoria/elation which had similarly low rates in both groups. Similarly, in the Baylor study in participants who had progressed to dementia, all NPS were more frequent in the moderate AD dementia group compared with mild AD dementia.

### Amyloid and tau assessments

The availability of neuroimaging and fluid biomarker data varied substantially across the cohorts (Table 2). The AIBL, BioFINDER-1, and MCSA studies routinely collected longitudinal data on amyloid PET as well as cerebrospinal fluid (CSF) Aβ_42_, pTau, and tTau in subsets of participants; CSF biomarker data were collected for over 90% of participants in BioFINDER-1. In contrast, biomarker data were not systematically collected in the Baylor study and French cohorts. Amyloid PET, but no other biomarkers, was assessed in a subset of participants in the 3C Bordeaux (49 non-demented participants, 4 AD participants) and AMI studies (61 non-demented participants) at 14 years and 7 years, respectively. Tau PET was only assessed in the BioFINDER-1 and MCSA cohorts.

**Table 2:** Availability of systematically collected biomarker data at baseline and/or follow-up in CONCORD-AD network cohorts

Biomarker	AIBL			BioFINDER-1			MCSA^a,d^	
	*CU*	*MCI*	*AD*	*CU^*b*^*	*MCI*	*AD*	*CU*	*MCI*	*AD*
*Measures of Aβ, n (%)*
PET	145 (12)^c^	36 (12)^c^	19 (6)^c^	272 (33)	169 (58)	N/A	1,592 (39)^c^	156 (28)^c^	N/A
CSF Aβ_42_	27 (2)	33 (11)	140 (44)	797 (96)	288 (99)	93 (100)	719 (18)^c^	64 (11)	N/A
*Measures of Tau, n (%)*
PET	N/A	N/A	N/A	52	42	2	579 (14)^c^	35 (6)	N/A
CSF pTau	27 (2)	33 (11)	140 (44)	797 (96)	288 (99)	93 (90)	719 (18)^c^	64 (11)	N/A
CSF tTau	27 (2)	33 (11)	140 (44)	797 (96)	288 (99)	93 (100)	719 (18)^c^	64 (11)	N/A

Aβ, amyloid-β; AD, Alzheimer’s disease; AIBL, Australian Imaging, Biomarkers & Lifestyle Flagship Study of Ageing; AMI, AGRICA-MSA-Institut fédératif de recherche en santé publique/Aging Multidisciplinary Investigation; BioFINDER-1, Biomarkers For Identifying Neurodegenerative Disorders Early and Reliably; CSF, cerebrospinal fluid; CU, cognitively unimpaired; MCI, mild cognitive impairment; MCSA, Mayo Clinic Study of Aging; N/A, not available; ND, non-demented; PET, positron emission tomography; pTau, phosphorylated tau; tTau, total tau; 3C Bordeaux, Three-City Study. ^a^In MCSA, PET and/or CSF biomarkers could be available at baseline and/or follow-up visits and not necessarily at the same study visit, as participants can select if they would like to undergo PET scans and/or lumbar puncture every time they visit the study. Follow-up visits occur every 15 months and neuroimaging studies are offered every 30 months or sooner if the participant’s cognitive status changes (e.g., progresses from CU to MCI). ^b^BioFINDER-1 CU group also included individuals with SCD. ^c^Longitudinal data available. ^d^MCSA did not actively follow-up participants diagnosed with dementia at study baseline.

## CHALLENGES OF CROSS-COHORT STUDIES

Although established AD cohorts offer a rich potential for increased understanding of AD risk factors, clinical heterogeneity, and disease progression, the challenges of cross-cohort studies must be examined.

### Establishing cohort studies

It is important to acknowledge that existing cohorts have not been established according to a uniform set of aims and using the same methods, and that individual cohort studies have unique hurdles to overcome. The design and conduct of cross-cohort studies must be conducted in accordance with regional and national guidelines, which vary not only with geography but also over time. Moreover, as longitudinal studies can be conducted for decades, there will be multiple protocol changes and adjustments needed to align with evolving guidelines. Cohort studies require sufficient infrastructure and funding to conduct testing over long intervals, meaning that expensive biomarker and/or imaging analyses may not be feasible, especially for large or geographically dispersed cohorts, or where biomarker characterization is not the primary purpose of the study. Study design can also be informed by previous studies and the evolving knowledge of AD, then tailored to meet new aims. For example, the Swedish BioFINDER-2 study launched in 2017 was designed to complement the pre-existing BioFINDER-1 study and address emerging issues regarding the role of tau pathology in different dementias and in their preclinical stages [66]. While BioFINDER-2 was not included in the current CONCORD-AD Network, potential future collaborations with this and other recently established longitudinal studies, could increase the breadth and depth of data in the future.

### Heterogeneous study designs

The CONCORD-AD collaboration model involves the sharing of independently conducted analyses to address key questions about disease progression, and patient-related outcomes in diverse patient populations, including both population- and community-based cohorts. This model facilitates evaluation of the impact of population and disease heterogeneity (e.g., geographic, educational, sociological, and cultural variations, study inclusion/exclusion criteria) on critical scientific questions. The included studies had a variety of aims, affecting study design and choice of assessments. This heterogeneity of assessments, while expected, limited the direct cross-comparison of cohorts and capacity for integration of patient-level data.

### Diagnostic challenges

Dementia diagnosis methodologies and thresholds for impairment also differed between studies and, notably, have evolved and improved since the start of some of the older studies such as PAQUID, which was established more than 10 years before some of the other cohorts included. Participants with dementia may now be diagnosed at earlier and milder stages compared with 30 years ago [67, 68].

The guidelines for diagnosis of MCI have also evolved over time and definitions differ between cohorts. In the CONCORD-AD network, prospective MCI groups were not assessed in the three French cohort studies, and either the Winblad [69] or Petersen criteria [70, 71] were used to define MCI in the remaining four cohorts. Although a potentially attractive idea, retrospective definition of an MCI group for cross-cohort comparison is problematic due to incomplete neuropsychological assessments, fluctuating cognition at visits, or missing information on comorbidities that may impact cognition. Diagnosing, and subsequently monitoring, MCI in a population-based study may be complex, particularly if the affected person does not report a cognitive complaint [72]. This may be compounded by social and cultural differences around what is felt to be “normal” for age, and perceived functional impairments associated with mild memory decline that may limit the reliability of caregivers to confirm problematic cognitive decline.

### Challenges associated with early detection in Alzheimer’s disease and dementia

International collaborations have the potential to leverage diverse clinical data in a variety of patient populations which ultimately could inform health-service planning for optimal early detection of cognitive decline. However, there are several challenges associated with early detection. Currently, in practice, AD is diagnosed with a variety of questionnaires that aim to measure memory function and neuroimaging tools, among other assessments [73]. The assessment of physical function is not routinely performed in practice, and thus an early-to-handle objective physical function test such as the Short Physical Performance Battery (SPPB) may provide useful prognostic information in the elderly population [74]. Additionally, differentiating between the normal course of aging versus the disease pathway seen in AD and dementia in the general population is an additional challenge for practitioners. Data suggest that screening for AD and related dementias in primary care to identify signs of early disease onset could allow for more timely intervention [75]. The CONCORD-AD network highlights the need for longitudinal tracking of at-risk individuals in order to provide early preventive interventions. Suitable, short cognitive screening tools for people thought to have MCI are lacking, e.g., the RAPCOG program aims to develop and validate screening tools for use in both MCI and dementia populations [76]. Digital assessments such as the Cogstate Brief Battery may be harnessed to develop registries of individuals at risk for AD and may inform use in the primary care setting [77].

### Comparing clinic- and community-based cohorts

Populations derived from specialist memory clinics differ from community cohorts as a result of differences in the demographics of individual participants, self-referral and healthcare professional-referral patterns, and differential access to healthcare services [78]. For example, more pronounced variations in MMSE were observed in CU participants in the population-based cohorts of MCSA, PAQUID, and 3C Bordeaux; notably, this variation was no longer observed in those with AD dementia. As for other forms of assessment, there is an inevitable disparity between cohorts due to inherent differences in study aims and designs.

The variability observed among cohorts highlights the impact of differing recruitment procedures and inclusion criteria. In clinical trials, with even more tailored inclusion and exclusion criteria, the trial population can greatly diverge from the original source population. By necessity, clinical trials often exclude those with comorbidities. This results in the recruitment of participants that may be healthier than the typical aging population, which could impact disease progression as captured by measures of cognitive and functional decline. Variations identified during cross-cohort comparisons could help further inform clinical trial design and recruitment in global clinical trials in order to include appropriate participants. These variations can also be mined for hypotheses related to the quantitative impact of methodologic or demographic difference on biomarkers or outcome measures.

### Data analysis

Cross-cohort comparisons also face challenges relating to the practical logistics of data sharing and data governance. While we can be mindful of differences in the study populations and timelines when looking at cross-cohort comparisons, it is not always easy or straightforward to combine data from different sources. The methodological challenges of analyzing and interpreting combined datasets can be lessened by describing the data for each cohort in detail, including the sources of the data, taking into account any specific underlying reasons for differences among cohorts, and clear communication from cohort investigators on potential caveats or limitations in the analysis and therefore subsequent interpretation of findings. The CONCORD-AD collaborative network eased difficulties in logistics surrounding data protection by sharing data summaries compiling population-level data for analysis rather than patient-level data, as shared in the GAAIN network.

## OPPORTUNITIES FOR CROSS-COHORT STUDIES

The primary advantage of the CONCORD-AD network is that it is a resource with a sufficiently large sample size to confirm or replicate findings of other studies and explore potential causes of heterogeneity in measures of interest, such as cognitive outcomes or rate of disease progression. Such a large cohort that includes patients across the wide spectrum of the AD disease course lends itself to be used for verification and replication of findings seen in smaller clinical trials. While data combined across the cohorts is not currently available, a potential future application of the CONCORD-AD network is harmonization of data to transparently integrate multiple pseudonymized data sources into a single federated database for use by researchers both within and outside the network. This could allow users to remotely access geographically dispersed data while ensuring data security and privacy.

Secondly, there are also research applications that could be addressed within collaboration projects in the future. This could include data collaboration comparing the CONCORD-AD network with the natural history of AD, from preclinical through clinical stages of disease. This continues to be a major research area of interest with key goals to increase understanding of: 1) cognitive and functional decline in slower and faster progressors along the AD continuum in globally representative cohorts; 2) the natural history of biomarker levels within the disease continuum; and 3) cognitive decline in the presence/absence of key biomarkers, vascular lesions, and comorbidities, and how these relate to major events within the AD continuum (e.g., dependency, institutionalization, death). All of these would improve our understanding of the multiple pathological processes leading to cognitive decline in AD and other non-AD-related dementias and processes underlying resilience to age-related disease processes [79, 80]. Thirdly, networks of cohort studies could also enable larger-scale characterization of a population of individuals at high risk for developing MCI and AD early in their disease course through the collection of longitudinal data in both cognitive and functional trajectories across disease stages. At present, there is varied consensus on the characterization of various AD subcategories, resulting in discrepancies in disease classification (i.e., MCI, prodromal AD) across clinical trials and in practice [81]. Comparing large-scale datasets across networks can improve our understanding of the core factors driving conversion from CU to MCI to AD and allow for improved disease classification with the aim of diagnosing patients earlier. Furthermore, data from highly harmonized cohorts could also provide an opportunity to quantify variance around a particular measure, such as annualized change in an assessment score, and to generate hypotheses regarding relative contributions of specific methodological differences to these variances.

This report raises awareness of the need to align cohort study designs in order to facilitate cross-cohort comparison, but further advances can also be made in analysis and interpretation of current data. Current biomarker data can be applied to analyses of core AD biomarkers, such as phosphorylated tau, to monitor disease progression [82]. Additionally, there are encouraging developments into a variety of other biomarkers that require validation [82]. Exploring existing biomarker data with different analytic techniques, such as using centiloid-based analyses to look at the degree of amyloid-positivity rather than binary amyloid-positive or -negative status, can enrich the information yielded by the cohorts. Additionally, investigating the potential utility of composite cognitive and functional endpoints in the CONCORD-AD network [16] and further subdivision of cognitive and functional measures may improve tracking of disease progression. By further exploring the changes in existing and novel biomarkers or clinical endpoints in large, collaborative studies, the data generated may improve our understanding of the clinical course of disease, to ultimately improve timely detection of AD and to better assess the effectiveness of interventions in AD.

## CONCLUSION

CONCORD-AD was created as an approach to mitigate the impact of AD on society, by bringing together global resources and expertise with the purpose of generating insights that can improve understanding of the natural history of the disease, inform design of clinical trials at all stages of the disease, and inform health-service planning for optimal patient access to new disease-modifying therapies once they become available. Expansion of these types of networks could be done to include other well-characterized and diverse cohorts representing a wide range of socioeconomic, ethnic, and geographic groups across the AD continuum, as well as closer alignment in data collection in future studies. This would further strengthen the research community’s potential to better understand, and eventually conquer, AD.

## Supplementary Material

Supplementary Material
