# Supplementary material for: Connecting Cohorts to Diminish Alzheimer’s Disease (CONCORD-AD): A Report of an International Research Collaboration Network
Source: J Alzheimers Dis. 2022 Jan 4;85(1):31–45. doi: 10.3233/JAD-210525 (PMC8842789; doi:10.3233/JAD-210525)
Supplement: Supplementary Material [file jad-85-jad210525-s001.pdf]

Supplementary Material

Connecting Cohorts to Diminish Alzheimer's Disease (CONCORD-AD): A Report of an International Research Collaboration Network

Supplementary Methods

Core dataset evaluation

Evaluation of the STROBE (STrengthening the Reporting of OBservational studies in Epidemiology) guidelines [1] and the available analyses from each cohort allowed a recommendation to be made for a "core dataset" to facilitate future cross-cohort analyses and ensure standardized descriptions of each cohort. Missing data/loss to follow-up were managed according to the methodology of each individual cohort. The core dataset comprises qualitative study information aligned to STROBE guidelines: a) study rationale, objectives, setting, participant recruitment, inclusion/exclusion criteria, clinical classification criteria as well as limitations and generalizability; b) quantitative demographic information: numbers of participants, follow-up period, attrition rates, age, gender, years of education, and apolipoprotein E ε4 allele carrier status; and c) where available, data on nine overlapping biomarkers, and clinical and cognitive measures of interest. The biomarkers included were amyloid positron emission tomography, hippocampal volume, cerebrospinal fluid Aβ (1–42) peptide, total tau, and phosphorylated tau. The clinical assessments analyzed were Clinical Dementia Rating – Sum of Boxes and Neuropsychiatric Inventory Questionnaire. The cognitive assessments utilized were the Mini-Mental State Examination (MMSE) and Alzheimer's Disease Assessment Scale – Cognitive Subscale delayed recall. In MCSA, MMSE was derived from the Short Test of Mental Status [2].

Supplementary Table 1. Definitions used for cognitive subgroups in each cohort

	AIBL	Baylor	BioFINDER-1	MCSA[a]	PAQUID	3C Bordeaux	AMI
Not Demented, CU, or Healthy elderly	Not meeting criteria for AD or MCI, answered no to the question "Do you have concerns about your memory?"	Normal Cognition: individuals with only a subjective memory complaint	Not demented: DSM-IV [3]. Healthy Elderly: normal performance on cognitive tests	Normal Cognition: consensus diagnosis. Participants who perform in the normative range and do not meet criteria for MCI or dementia	Not demented: DSM-III-R [4]	Not demented: DSM-IV [3]	Not demented: DSM-IV [3]
MCI	Winblad criteria [5][b]	Petersen criteria (1999) [6][c]	Petersen criteria (2004) [7][d]	Petersen criteria (2004) [7][e]	Not assessed[f]	Not assessed[f]	Not Assessed[f]
AD	NINCDS-ADRDA criteria (1984) [8]	NINCDS-ADRDA criteria (1984) [8]	NINCDS-ADRDA criteria (2011) [9]	DSM IV dementia [3], NINCDS-ADRDA criteria (1984, 2011) [8,9][g]	NINCDS-ADRDA criteria (1984) [8]	NINCDS-ADRDA criteria (1984) [8]	NINCDS-ADRDA criteria (1984) [8]

AD, Alzheimer's disease; AMI, AGRICA-MSA-Institut fédératif de recherche en santé publique/Aging Multidisciplinary Investigation; AIBL, Australian Imaging, Biomarkers & Lifestyle Flagship Study of Ageing; Baylor, Alzheimer's Disease and Memory Disorders Center at Baylor College of Medicine; BioFINDER-1, Biomarkers For Identifying Neurodegenerative Disorders Early and Reliably-1; CDR, Clinical Dementia Rating; CU, cognitively unimpaired; DSM-III-R, Diagnostic and Statistical Manual of Mental Disorders (Third Edition - Revised); DSM-IV, Diagnostic and Statistical Manual of Mental Disorders (Fourth Edition); ICD-10, 10th revision of the International Statistical Classification of Diseases and Related Health Problems; MCSA, Mayo Clinic Study of Aging; MCI, mild cognitive impairment; NINCDS-ADRDA, National Institute of Neurological and Communicative Disorders and Stroke and the Alzheimer's Disease and Related Disorders Association; NINDS-AIREN, National Institute of Neurological Disorders and Stroke and the Association Internationale pour la Recherche et l'Enseignement en Neurosciences; PAQUID, Personnes Agées QUID; SD, standard deviation; 3C Bordeaux, Three-City Study. [a] At a weekly-held conference, three evaluators (the nurse or study coordinator, the physician, and a neuropsychologist) discuss all the information for each participant and make the final diagnosis (i.e., CU, MCI, dementia) by consensus [b] Participants presenting with a clinical diagnosis of MCI were further required to demonstrate a score of ≥1.5 SD below the age-adjusted mean on ≥1 neuropsychological task in order to be retained in the MCI category. [c] Required CDR 0.5, subjective memory loss and no other domains of involvement but also required neuropsychological testing to verify the memory disorder. [d] MMSE 24–30 with neuropsychological battery to stratify SCD from MCI [e] Cognitive concern expressed by a physician, informant, participant, or nurse; cognitive impairment in ≥1 domains; essentially normal functional activities; not demented. [f] MCI criteria published by Petersen et al. were retrospectively applied for specific analyses, but no active definition of MCI was used in the PAQUID, 3C Bordeaux, or AMI cohorts. [g] Dementia (including AD).

Supplementary Figure 1. Comparison of baseline CDR-SB scores in available cohorts in (A) CU, (B) MCI, and (C) AD participants

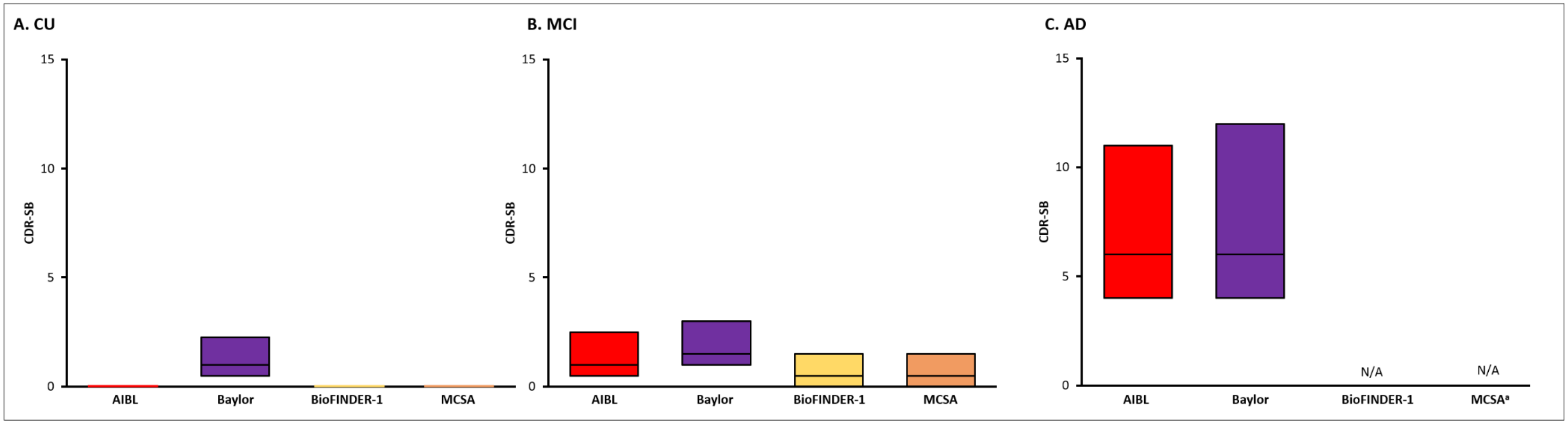

AD, Alzheimer's disease; AIBL, Australian Imaging, Biomarkers & Lifestyle Flagship Study of Ageing; Baylor, Alzheimer's Disease and Memory Disorders Center at Baylor College of Medicine; BioFINDER-1, Biomarkers For Identifying Neurodegenerative Disorders Early and Reliably; CDR-SB, Clinical Dementia Rating–Sum of Boxes; CU, cognitively unimpaired; MCSA, Mayo Clinic Study of Aging; ND, no data. [a] The AD group in MCSA included dementia of various causes.

REFERENCES

[1] Von Elm E, Altman DG, Egger M, Pocock SJ, Gøtzsche PC, Vandenbroucke JP, STROBE Initiative (2014) The Strengthening the Reporting of Observational Studies in Epidemiology (STROBE) Statement: Guidelines for reporting observational studies. *Int J Surg* **12**, 1495-1499.

[2] Kokmen E, Smith GE, Petersen RC, Tangalos E, Ivnik RC (1991) The short test of mental status: Correlations with standardized psychometric testing. *Arch Neurol* **48**, 725-728.

[3] American Psychiatric Association (1994) *Diagnostic and Statistical Manual of Mental Disorders (Fourth Edition): DSM-IV*, American Psychiatric Association (APA), Washington, DC.

[4] American Psychiatric Association (1987) *Diagnostic and Statistical Manual of Mental Disorders (Third Edition - Revised): DSM-III-R,* American Psychiatric Association (APA), Washington, DC.

[5] Winblad B, Palmer K, Kivipelto M, Jelic V, Fratiglioni L, Wahlund LO, Nordberg A, Bäckman L, Albert M, Almkvist O, Arai H, Basun H, Blennow K, de Leon M, DeCarli C, Erkinjuntti T, Giacobini E, Graff C, Hardy J, Jack C, Jorm A, Ritchie K, van Duijn C, Visser P, Petersen RC (2004) Mild cognitive impairment--beyond controversies, towards a consensus: Report of the International Working Group on Mild Cognitive Impairment. *J Intern Med* **256,** 240-246.

[6] Petersen RC, Smith GE, Waring SC, Ivnik RJ, Tangalos EG, Kokmen E (1999) Mild cognitive impairment: Clinical characterization and outcome. *Arch Neurol* **56**, 303-308.

[7] Petersen RC (2004) Mild cognitive impairment as a diagnostic entity. *J Intern Med* **256**, 183-194.

[8] McKhann G, Drachman D, Folstein M, Katzman R, Price D, Stadlan EM (1984) Clinical diagnosis of Alzheimer's disease: Report of the NINCDS-ADRDA Work Group under the auspices of Department of Health and Human Services Task Force on Alzheimer's Disease. *Neurology* **34**, 939-939.

[9] McKhann GM, Knopman DS, Chertkow H, Hyman BT, Jack Jr CR, Kawas CH, Klunk WE, Koroshetz WJ, Manly JJ, Mayeux R (2011) The diagnosis of dementia due to Alzheimer's disease: Recommendations from the National Institute on Aging-Alzheimer's Association workgroups on diagnostic guidelines for Alzheimer's disease. *Alzheimers Dement* **7**, 263-269.
